# Gene expression profiling to identify eggshell proteins involved in physical defense of the chicken egg

**DOI:** 10.1186/1471-2164-11-57

**Published:** 2010-01-21

**Authors:** Vincent Jonchère, Sophie Réhault-Godbert, Christelle Hennequet-Antier, Cédric Cabau, Vonick Sibut, Larry A Cogburn, Yves Nys, Joel Gautron

**Affiliations:** 1 INRA, UR83 Recherches Avicoles, F-37380 Nouzilly, France; 2 Department of Animal and Food Sciences, University of Delaware, Newark, DE 19717 USA; 3 Institut Technique Avicole, F-37380 Nouzilly, France

## Abstract

**Background:**

As uricoletic animals, chickens produce cleidoic eggs, which are self-contained bacteria-resistant biological packages for extra-uterine development of the chick embryo. The eggshell constitutes a natural physical barrier against bacterial penetration if it forms correctly and remains intact. The eggshell's remarkable mechanical properties are due to interactions among mineral components and the organic matrix proteins. The purpose of our study was to identify novel eggshell proteins by examining the transcriptome of the uterus during calcification of the eggshell. An extensive bioinformatic analysis on genes over-expressed in the uterus allowed us to identify novel eggshell proteins that contribute to the egg's natural defenses.

**Results:**

Our 14 K Del-Mar Chicken Integrated Systems microarray was used for transcriptional profiling in the hen's uterus during eggshell deposition. A total of 605 transcripts were over-expressed in the uterus compared with the magnum or white isthmus across a wide range of abundance (1.1- to 79.4-fold difference). The 605 highly-expressed uterine transcripts correspond to 469 unique genes, which encode 437 different proteins. Gene Ontology (GO) analysis was used for interpretation of protein function. The most over-represented GO terms are related to genes encoding ion transport proteins, which provide eggshell mineral precursors. Signal peptide sequence was found for 54 putative proteins secreted by the uterus during eggshell formation. Many functional proteins are involved in calcium binding or biomineralization--prerequisites for interacting with the mineral phase during eggshell fabrication. While another large group of proteins could be involved in proper folding of the eggshell matrix. Many secreted uterine proteins possess antibacterial properties, which would protect the egg against microbial invasion. A final group includes proteases and protease inhibitors that regulate protein activity in the acellular uterine fluid where eggshell formation takes place.

**Conclusions:**

Our original study provides the first detailed description of the chicken uterus transcriptome during formation of the eggshell. We have discovered a cache of about 600 functional genes and identified a large number of encoded proteins secreted into uterine fluid for fabrication of the eggshell and chemical protection of the egg. Some of these uterine genes could prove useful as biological markers for genetic improvement of phenotypic traits (i.e., egg and eggshell quality).

## Background

**Figure 1:**
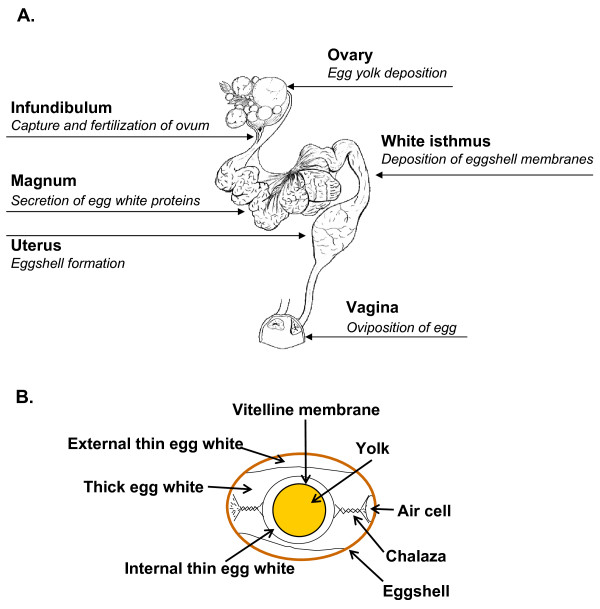
**Chicken oviduct segments (A) and egg components (B)**.

The chicken egg is formed in the hen's left ovary and oviduct. The ovary supports the accumulation of egg yolk proteins and maturation of the ovum (Figure 1A). After ovulation, the yolk enters the oviduct, where albumen, eggshell membranes and the eggshell are sequentially deposited in the different segments of the hen's reproductive tract (magnum, white isthmus and uterus, respectively) (Figure 1). The hen manufactures a cleidoic egg [1], which is a completely self-sufficient and aseptic biological package for the extra-uterine development of the avian embryo. This adaptation implies that the egg must contain all components required for the complete extra-uterine development of a fertilized ovum into a viable chick in 21 days. To ensure this dynamic challenge, the egg must possess a broad range of biological activities and natural defenses [2,3]. The avian egg contains vitamins, minerals and proteins (albumen and yolk), yolk lipids and calcium salts (eggshell) necessary for the development of the embryo. Furthermore, the chicken and egg have been an important basic food for humans worldwide for millennia. The egg has a high nutritive value from a well-balanced source of amino acids that are easily assimilated [4]. When faced with physical and/or microbial aggression, the egg has two major defensive mechanisms--a chemical protection system composed of yolk, albumen and eggshell matrix proteins that provide antimicrobial protection [2,3,5,6], and the intact eggshell that acts as a physical barrier to protect against bacterial invasion [6,7].

**Figure 2:**
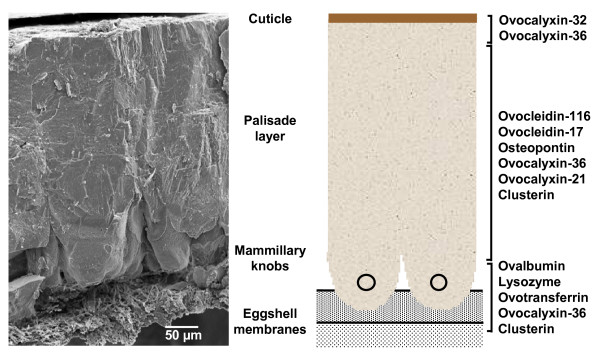
**Cross section of the eggshell and distribution of known matrix proteins**.

The eggshell itself is a complex bioceramic material formed in the uterus (shell gland) segment of the chicken's oviduct. It consists of inner and outer eggshell membranes, an intermediate calcified zone composed of mammillary and palisade layers, and an outer cuticle layer (Figure 2). Organic components and ions required for eggshell mineralization are secreted by the uterus into the acellular milieu of uterine fluid, which bathes the egg during its 20 hour travel through the hen's oviduct. The eggshell is composed of calcium carbonate (polycrystalline calcite) deposited onto the eggshell membranes that are pervaded with organic matrix, which itself is a complex mixture of proteins, glycoproteins and proteoglycans [8,9]. The organic matrix plays a major role in assembly of the bioceramic layer and in determination of its mechanical properties. Therefore, identification of the protein complement of the uterus is the first step toward a more complete understanding of the diverse biological functions of the avian eggshell.

Matrix proteins are traditionally studied using a variety of biochemical and molecular techniques. These classical approaches have allowed identification of ten proteins (Figure 2) that belong to three functional groups. Firstly, three egg white proteins, ovalbumin [10], lysozyme [11] and ovotransferrin [12] are found in the eggshell. Secondly, eggshell contain ubiquitous proteins including osteopontin, a phosphorylated glycoprotein present in bone and other hard tissues [13], and clusterin, a secretory glycoprotein that is also found in the egg white [14]. Thirdly, several matrix proteins are unique to shell calcification and only secreted in regions of the oviduct where eggshell calcification occurs. Ovocleidin-17 (OC-17) was the first eggshell protein purified from the shell [15]. This secretory protein (OC-17) is a C-type, lectin-like phosphoprotein [16] that occurs in glycosylated (23 kDa) and nonglycosylated (17 kDa) forms in the shell matrix [17]. Ovocleidin-116 (OC-116) was the first eggshell matrix protein to be cloned [18]. OC-116 forms the protein core of a 120-200 kDa dermatan sulfate proteoglycan called ovoglycan [19,20], which is found throughout the compact calcified eggshell [18]. Ovocalyxin-32 (OCX-32), a 32 kDa uterine-specific protein, is concentrated in the outer calcified region and in the cuticle of the calcified shell [21]. Ovocalyxin-36 (OCX-36) is a 36 kDa protein found only in the shell gland (uterus) where eggshell calcification takes place [22]. Uterine OCX-36 message levels are strongly up-regulated during eggshell calcification. OCX-36 is predominantly localized in the inner part of the shell and homologous to innate immune response proteins [22]. Ovocalyxin-21 (OCX-21) is another eggshell specific protein that was recently cloned and characterized [8].

Although many major proteins in the egg have been identified, we need a more complete and detailed picture of the genes encoding all proteins required for eggshell formation. The availability of the chicken genome sequence [23] and recent development of high-throughput genomic and proteomic assays provide powerful tools for a more complete characterization of egg components [24]. A major advance in understanding the complex nature of the eggshell and its assembly in the hen's oviduct came from the work of Mann *et al*. [25,26], who used a focused proteomics approach to identify 528 proteins contained within the eggshell.

The present study provides an original description of the oviduct transcriptome in the laying hen and a repertoire of functional genes that are highly expressed in the uterus during eggshell calcification. Our approach provides the first global description of highly expressed uterine genes and their putative secretory proteins that are deposited in the eggshell. These functional components ensure proper eggshell formation, which provides a natural physical barrier and robust antimicrobial protection for the developing chick embryo or the edible egg.

## Results

### Identification of uterine specific genes

**Figure 3:**
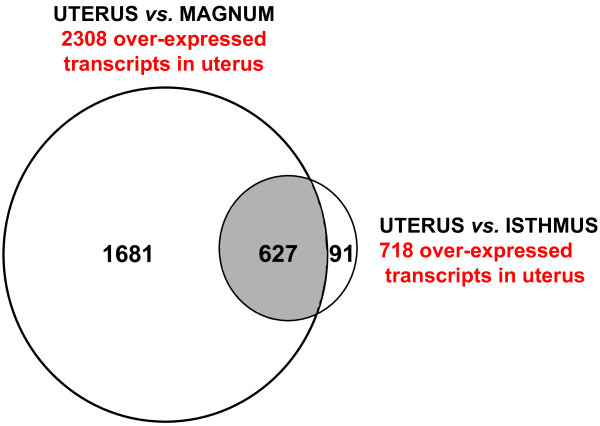
**Venn diagram of over-expressed genes in the uterus compared with the magnum and white isthmus**.

We have used our custom Del-Mar 14 K Chicken Integrated Systems microarray [27] to analyze gene expression in different segments of the hen's oviduct during formation of the eggshell. Oviducal tissue samples were collected at 18 hr post ovulation from the magnum (where egg white proteins are secreted), the white isthmus (where inner and outer eggshell membranes are deposited) and the uterus (where eggshell calcification occurs). A total of 2308 genes were over-expressed [false discovery rate (FDR) <0.05] in the uterus when compared to the magnum (Ut/Ma; Figure 3). When global gene expression in uterus was compared to that of the white isthmus (Ut/Wi), 718 genes were over expressed in uterus. We found 1681 over-expressed uterine transcripts that were unique to the Ut/Ma contrast and 91 over-expressed uterine transcripts that were unique to the Ut/Wi contrast (Additional file 1). A total of 627 highly expressed uterine transcripts were common between the two contrasts [uterus *versus *the magnum (Ut/Ma) or the uterus versus the white isthmus (Ut/Wi)], which indicates that these uterine genes are highly expressed in the hen's oviduct during calcification of the egg.

The Del-Mar 14 K chicken cDNA microarray is composed of 18,230 cDNA inserts, which correspond to 14,053 unique genes. These cDNA were selected to represent an integration of four physiological systems (metabolic, somatic, neuroendocrine and reproductive systems) from our collection of ~40 K EST clones [28]. Our array represents 14,049 contigs and 3,716 singlets from our original assembly of a chicken gene index [29]. Consequently, there is some redundancy of genes represented on the array, where the 627 uterine transcripts corresponded to 605 unique cDNA sequences (Additional file 1). If we raise the significance threshold to greater than 1.4-fold difference, 440 genes were over-expressed in the uterus compared to the magnum, whereas 202 transcripts were higher in the uterus than the white isthmus. The number of genes over-expressed still remains high even if we consider a greater than 2-fold change as cut-off, where 165 transcripts were over-expressed in the uterus compared to magnum and 29 transcripts expressed higher in the uterus than the white isthmus.

### Verification of gene expression by qRT-PCR analysis

**Figure 4:**
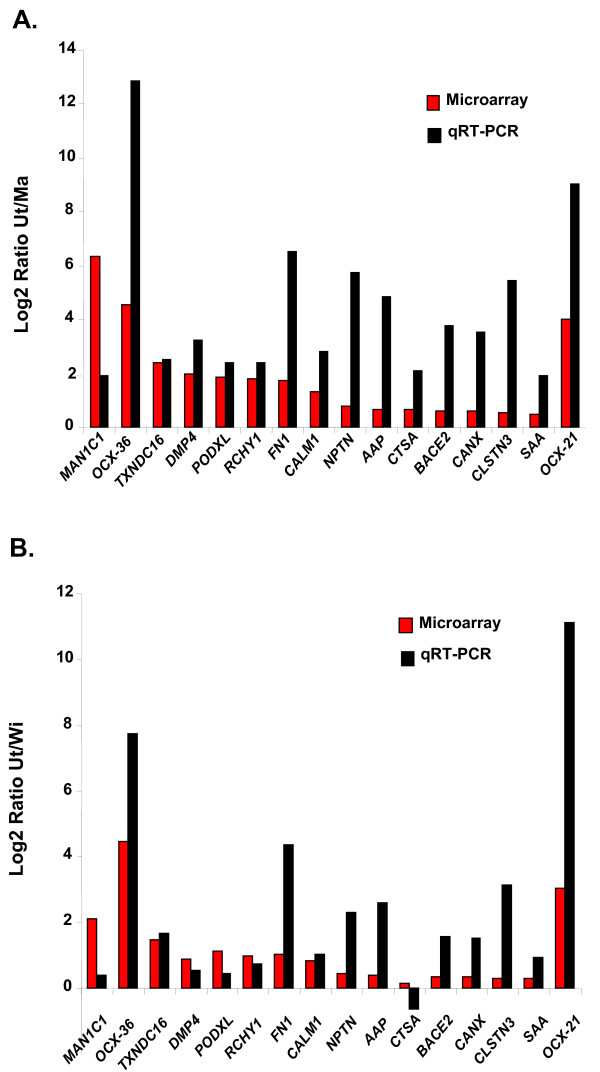
**Comparison of gene expression in the hen's oviduct from microarray and qRT-PCR analyses**.

Of 605 genes that were over-expressed in the uterus by microarray analysis, 16 genes were selected for verification of transcript abundance using quantitative real time PCR (qRT-PCR) (Figure 4). These 16 genes were chosen to represent a wide range of gene expression (0.1 to 6.3 log2 ratio). Normalized expression levels of genes over-expressed in the uterus were compared to that of the magnum and white isthmus. Log2 ratios of gene expression [determined by qRT-PCR analysis in the uterus *versus *magnum (Ut/Ma) or the uterus *versus *white isthmus (Ut/WI)] were compared to expression levels obtained using microarray analysis. Over-expression of these genes in the uterus was confirmed by qRT-PCR analysis for 31 of the 32 measurements. However, the expression of cathepsin A (*CTSA*) was slightly lower in the uterus compared to isthmus, although the microarray data showed slightly higher (10%) expression in the uterus. In the majority of cases, the amplitude of gene expression was higher with qRT-PCR analysis than with microarray analysis. However, the amplitude was lower for mannosidase (*MAN1C1*) in both contrasts (Ut/Ma and Ut/WI), while dentin matrix protein-4 (*DMP4*), podocalyxin (*PODXL*), and zinc finger protein 363 (*RCHY1*) were lower in the Ut/WI contrast. The qRT-PCR analysis confirmed that expression of 15 genes selected for verification was significantly higher (P < 0.05) in the uterus when compared to the magnum (Additional file 2). When compared to isthmus, uterine expression was higher (*P *< 0.05) for three other genes: ovocalyxin-36 (*OCX-36*), alpha-2-antiplasmin (*AAP*) and ovocalyxin-21 (*OCX-21*). Although the abundance of 18S RNA from each tissue was not significantly different, the normalization process increased variability of gene expression across three tissues. This variability could explain the absence of statistical differences for other genes in the uterus *versus *white isthmus contrast. Nevertheless, microarray analysis shows many genes over-expressed in the uterus when compared to either the magnum or white isthmus.

### Functional annotation of uterine-specific genes

The 605 uterine gene sequences were annotated with assembled contigs and singletons and compared to translated proteins in public databases. As a first approach, we used the bioinformatics pipeline developed by Système d'Information d' Analyse du GENome des Animaux d'Elevage (SIGENAE) [30]. The SIGENAE EST assemblies produce contigs from partial cDNA sequences found in public databases. The 605 over-expressed uterine transcripts correspond to 537 chicken contigs present in the SIGENAE database. Among these, some contig sequences were redundant and after removal of the redundancy, 500 unique transcripts were identified. These 500 transcripts correspond to 469 unique chicken UniGene entries [31]. The 55 remaining sequences have no hits in the UniGene database and therefore correspond to unknown genes that are differentially expressed in the uterus of the laying hen.

The SIGENAE Bioinformatic tools were also used to identify proteins encoded by these uterine transcripts. Similarity searches between contig sequences and UniProtKB entries were performed with an expected (E) value of 10^-5 ^as threshold. We found that the 605 transcripts highly expressed in the uterus were related to 437 proteins with a unique UniProtKB ID. Among these, 90 were chicken (*Gallus gallus*) proteins, while three additional proteins were issued from other birds (duck, turkey and pheasant). A large majority of proteins was identified by homology to human (161), mouse (64), rat (26), bovine (25), other mammalian species (48 proteins), or other species (20).

### Gene Ontology (GO) term enrichment of uterine genes

**Figure 5:**
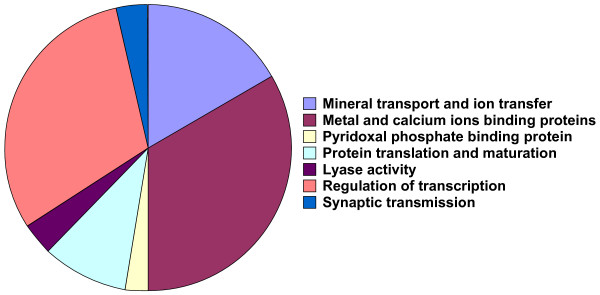
**Piechart showing distribution of over-expressed uterine transcripts according to potential biological properties**. EASE was used to determine GO terms (biological process and molecular function), enriched in the uterine transcriptome and classified in eight distinct groups according to their potential biological functions (see Table 1).

Gene Ontology (GO) terms are widely used for global interpretation of the function of proteins encoded by genes revealed by microarray analysis. Expression Analysis Systematic Explorer (EASE) software [32] was used to compare GO terms significantly enriched in the uterus transcriptome by comparison to the total GO terms represented on the Del-Mar 14 K cDNA microarray. GO terms were assigned with the best EASE score (a modified Fisher Exact *P*-Value) and high enrichment value (He). The GO terms were then classified in various groups according to biological functions (Table 1; Figure 5).

**Table 1:** Gene ontology (GO) term enrichment in the uterine transcriptome

Description	GO terms*	Enrichment	EASE score	Gene symbol
**Mineral transport and ion transfer**	F-0046933	7.91	0.00018	ATP5B, ATP6V1E1, ATP5A1, ATP5G1
	P-0015986	7.69	0.00021	ATP5B, ATP6V1E1, ATP5A1, ATP5G1
	F-0046961	6.50	0.00056	ATP5B, ATP6V1E1, ATP5A1, ATP5G1
	F-0016820	7.80	0.00077	ATP5A1, ATP1A1, ATP2A2
	P-0006811	3.21	0.00650	ATP5B, WNK1, P2RX4, ATP5A1, ATP1B1, GRIN1, ADD1, SCNN1A
	C-0016469	5.80	0.00093	ATP5B, ATP6V1E1, ATP5A1, ATP5G1
	P-0006814	5.49	0.01166	ATP1A1, SCNN1G, ATP1B1, SCNN1A
	P-0006810	1.73	0.04275	ANT3, SLC6A8, CYC, HIAT1, ATP1A1, UQCRFS1, P2RX4, CLCN2, GLRB, ATP2C2, ENSA, HBB, GDA
**Metal and calcium ions binding proteins**	F-0046872	1.67	0.02991	ADD3, ATP5B, CYC, BRE1A, ATP5A1, HBB, PPP2CA, WDFY1, UQCRFS1, TYRP2, PEPD, HMOX1_CHICK, RCHY1, RNF114, ZC3H11A, ADD1, SOD1, DNAJA2, RPL37A
	F-0005509	1.90	0.00701	DTNB, CANX, MAN1C1, ATP2A2, CALM1, MACF1, STAT3, HSP90B1, SLIT2, CALM2, SLIT3, NECAB3, CAMKK2, MEGF6, FKBP9, DSG2, NUCB2, ANXA2, CUBN, CLSTN3
**Pyridoxal phosphate binding**	F-0030170	4.46	0.01013	SGPL1, GOT1, YCBX
**Protein translation and maturation**	F-0005198	2.31	0.01017	ADD3, TUBB2C, GAG, TUBA1C, KRT8, TBB2, TBA5, ADD1, PNN, GFAP, ACTR1A
	P-0051258	4.71	0.01998	TUBB2C, TUBA1C, TBB2, TBA5
	C-0043234	3.05	0.02549	TUBA1C, TBB2, MYH9, TBA5, TUBB2C, CDCA8
**Synaptic transmission**	P-0007268	4.71	0.01998	NPTX2, GRIN1, PI4KA, STX1A
**Endoplasmic reticulum**	C-0005783	1.75	0.02295	INSIG1, CANX, PPGB, RTN3, NECAB3, CALR, ALG3, TTC35, ADFP, D17WSU104E, SCAP, SURF4, EMID1, PTPN1, HSP90B1, HSPA5, HMOX1
**Lyase activity**	F-0016829	3.32	0.03303	ACLY, SGPL1, ODC1, AMD1
**Cytosol**	C-0005829	1.72	0.03740	PIK3R1, MVD, PDCD6IP, PPP2CA, EZR, WDFY1, ODC1, OGT, CEP290, TRPC4AP, PACSIN2, HEBP1, MYH9, ARFGAP3, SEC14L2, NUCB2
**Regulation of transcription**	P-0045449	2.48	0.04107	PPP2CA, MAD4, STAT3, BTG1, MLX, ID2, FUSIP1
	F-0000166	1.46	0.02473	ATP5B, GNAI2, CIRBP, MAPK06, GRP78, TBB2C, ATP5A1, HSPA8, ATP1A1, DDX5, TUBA1C, PRKAR1A, ATP2A2, HNRPH3, TUBB2C, PFKP, SGK1, TBB2C, ITPK1, SARS, ARF1, HNRPQ, MYH9, TRA2B, HNRPD, PPARGC1A, CKB, MTHFD1

*GO term categories: Cellular component (C), Biological process (P) and Molecular function (F). GO term enrichment was analyzed using EASE software http://david.abcc.ncifcrf.gov/ease.

The most prominent proteins are involved in mineral transport and ion transfer in the uterus during formation of the eggshell. The terms hydrogen ion transporting ATP synthase activity, rotational mechanism (GO:0046933), ATP synthesis coupled proton transport (GO:0015986), hydrogen ion transporting ATPase activity, rotational mechanism (GO:0046961), and proton-transporting two-sector ATPase complex (GO:0016469) are related to proteins controlling ATPase activity and encode four different ATP synthases. This group also included the GO:0016820 (Hydrolase activity, acting on acid anhydrides, catalyzing transmembrane movement of substances) composed of six genes coding an ATP synthase, a sodium/potassium transporting ATPase and a sarcoplasmic/endoplasmic reticulum calcium ATPase. Nine genes refer to ions transport (GO:0006811), encoding alpha-adducin (ADD1), [a membrane-cytoskeleton associated protein], purinergic receptor P2Y (P2RX4), [a receptor for ATP], a glutamate (NMDA) receptor subunit zeta-1 (GRIN1), [a glutamate-gated ion receptor], a serine/threonine-protein kinase (WNK1), which controls sodium and ion transport, sodium/potassium-transporting ATPase, the ameloride-sensitive sodium channel subunit alpha (SCNN1). Sodium ion transport term (GO: 0006814) include sodium/potassium-transporting ATPase alpha (ATP1A1) and beta (ATP1B1) and the ameloride-sensitive sodium channel subunits alpha (SCNN1A) and gamma (SCNN1G). Finally, the term GO: 0006810, contains 16 genes encoding 14 transport proteins, which include ADP/ATP translocase 3 (ANT3), probable calcium transporting ATPase (ATP2C2), sodium/potassium-transporting ATPase (ATP1A1), a sodium- and chloride-dependent creatine transporter (SLC6A8) and several miscellaneous proteins.

Another group of importance was composed of uterine genes encoding ion binding proteins, which are essential for the mineral phase interactions during eggshell calcification. Forty genes encode 19 proteins that selectively interact with metal ions (GO: 0046872), and 20 calcium ion binding proteins (GO: 0005509). Among this last group of calcium ion binding proteins, annexin (ANXA2), desmoglein-2 (*DSG2*), EGF-like domain-containing protein (MEGF6) and mannosidase (MAN1C1) transcripts were highly expressed in the hen's uterus during egg shell calcification. These genes had the highest expression levels, which were 3.3- to 8.9-times higher than the mean normalized intensity of all uterine genes. It is notable that amongst the 605 uterine specific transcripts, MAN1C1 was the most abundant normalized intensity and the greatest difference (79.4-fold) compared to the two other oviduct segments (magnum and white isthmus). Another group of binding proteins includes three different proteins, which interact selectively with biologically-active vitamin B6 (GO: 0030170 - Pyridoxal phosphate binding).

GO terms for structural molecule activity (GO: 0005198), protein polymerization (GO: 0051258), and protein complexes (GO: 0043234) are related to protein translation, maturation and post-translational modifications. Lyase activity (GO: 0016829) is related to proteins, which catalyze cleavage of C-C, C-O, C-N and other bonds by ways other than hydrolysis or oxidation. Five genes encode four different proteins related to synaptic transmission and nervous control of uterine activity. Finally, two terms (GO: 0045449 - regulation of transcription, GO: 0000166 - nucleotide binding), are composed of 41 transcripts corresponding to 35 different proteins involved in regulation of gene transcription.

### Putative secreted eggshell proteins

Our cDNA microarray analysis has identified 605 highly-expressed uterine transcripts. The next hurdle is to determine which genes encode the numerous biologically-active proteins secreted by the hen's uterus during eggshell formation. Genes encoding uterine proteins can be divided in two general groups: [1] intracellular proteins involved in metabolism of the uterus and transporters of mineral precursors for the eggshell, but not secreted into the oviduct lumen and [2] the extra-cellular proteins, which are secreted into the oviduct and deposited in the eggshell. To solve this problem, we have examined the eggshell "secretome". Our initial approach was a comparison of the translated protein sequences from the 605 over-expressed uterine transcripts (contig sequences) with the eggshell proteins identified by recent proteome surveys [25,26]. A total of 52 genes over-expressed in uterus encode proteins revealed by the earlier proteomic analysis (Additional file 3). In a second approach, the 437 proteins derived from the uterine genes were analyzed using SignalP [33] to evaluate the presence of a signal peptide sequence required for protein secretion.

Based on the presence of conserved signal sequences, we estimated that about 14% of the genes over-expressed in the uterus encode 54 proteins possessing a signal peptide. The potential function of these 54 proteins was examined by bioinformatic analysis using multiple genomic resources (UniProtKB/Swiss-Prot, Pfam, PROSITE and InterPro databases) (Table 2). Finally, the isoelectric point and amino acid composition were determined *in silico *to establish which ones are negatively charged in the uterine fluid, since this property would favor an interaction with the calcite crystal during the calcification process. The biochemical properties [predicted acidic (<5.5) basic (>8.5) and isoelectric point (pI)] of some putative secreted proteins are presented in Table 3. We found nine basic proteins and a higher proportion of acidic proteins (i.e., 24 proteins have a pI ranging from 3.21 to 5.47). Among the acidic proteins, calnexin (CANX; pI = 4.47)), endoplasmin (HSP90B1; pI = 4.81), peptidyl-prolyl cis-trans isomerase (FKBP9; pI = 4.82), nucleobindin (NUCB2; pI = 4.99), follistatin-related protein 1 (FSTL1; pI = 5.15) and calsyntenin-3 (CLSTN3; pI = 5.17) have calcium binding properties. The acidic group also contains three proteins with immunoglobulin-like domains: neuroplastin (NPTN; pI = 3.21), ICOS ligand (ICOSLG; pI = 5.15) and beta-2-microglobulin (B2M; pI = 5.46). Two additional proteins have antimicrobial properties [ovocalyxin-36 (OCX-36; pI = 5.38) and avian β-defensin 9 (DEFB9)]. Finally, beta-amyloid protein (APP; pI = 4.65) and tissue factor pathway inhibitor 2 (TFPI2; pI = 9.06) are known inhibitors of serine proteinases.

**Table 2:** Functional annotation of putative proteins secreted in the hen's uterus

Protein name (Accession #)	Functional annotation
Ovocalyxin-21 (IPI00574331)	Eggshell specific protein containing a Brichos domain

Podocalyxin (O00592)	Sialoprotein highly negative charged containing a podocalyxin (CD34 antigen) domain. Involved in renal filtration, associated with cancers

Metalloproteinase inhibitor 2 (TIMP2) (O42146)	Metalloproteinase inhibitor, NTR (netrin) domain. Tissue remodelling, complexes with metalloproteinases and irreversibly inactivates them

Lysosomal alpha-mannosidase (O46432)	Alpha mannosidase middle domain, Glycosyl hydrolases family 38 N- and C-terminal domains, which participate in the maturation of N-glycans

ICOS ligand (O75144)	Proteins with domains analog to immunoglobulins (Ig-like C2-type, Ig-like V-type), immunoglobulin superfamily. BTN/MOG family. Important for protein folding

Slit homolog 3 and 2 proteins (O88280; O94813)	Embryonic development and calcium-binding protein containing CTCK (C-terminal cystine knot-like), Epithelial Growth Factor (EGF)-like, laminin-like LRR (leucine-rich) repeat, Calcium-binding EGF-like domains

L-dopachrome tautomerase (O93505)	Involved in the formation of pigments, binds 2 copper ions (Common central domain of tyrosinase)

Serum amyloid A protein (P02740)	Apolipoprotein of the HDL complex. Major acute phase reactant protein containing a serum amyloid A protein domain

Tissue alpha-L-fucosidase (P04066)	Alpha-L-fucosidase putative active site involved in glycoprotein metabolism

Endoplasmin (P08110)	Molecular chaperone that functions in the processing and transport of secreted proteins. Contain ATP-binding region, calcium ion binding, heat shock protein 90 (HSP90)-like ATPase domains

Apolipoprotein A-I (P08250)	Lipid transport protein. Apolipoprotein A1/A4/E domain

Alpha-2-antiplasmin (P08697	Serine protease inhibitor (serpin)

Lysosomal protective protein (P10619)	Protective protein with a carboxypeptidase activity

Beta-2-microglobulin (P21611)	Essential subunit of major histocompatibility complex class I molecules containing Ig-like domains.

Osteopontin (P23498)	Glycoprotein of bone, eggshell, kidney and various body secretions. Involved in the mineralization of the shell

Neuronal pentraxin-2 (P47972)	Calcium binding protein involved in acute immunological responses. Contains a concanavalin-A lectin domain.

Glioma pathogenesis-related protein 1 (P48060)	Belongs to the CRISP family. Contain allergen V5/Tpx-1 related, SCP-like extracellular (Ca++ chelating serine protease) domains

Glycine receptor subunit beta (P48167)	Neurotransmitter-gated ion channel. Increase chloride conductance

Receptor-type tyrosine-protein phosphatase-like N (P56722)	Implicated in neuroendocrine secretory processes

Amyloid beta A4 protein (P79307)	Protein associated with Alzheimer disease containing an heparin-binding and Kunitz/bovine pancreatic trypsin inhibitor domains

Nucleobindin-2 (P80303)	Calcium binding proteins containing an EF-hand domain

Neuroplastin (P97300)	Cell adhesion molecule of the immunoglobulin superfamily. It contains Ig-like domains (Ig-like C2 and V types)

Golgi apparatus protein 1 (Q02391)	Receptor for Fibroblast Growth Factors (FGF) containing cysteine-rich GLG1 repeat

Follistatin-related protein 1 (Q12841)	Involved in cell proliferation, differentiation and survival. Containing calcium-binding EF-hand, a protease inhibitor, kazal type and von Willerbrand factor domains

FK506-binding protein 9 (Q2KJC8)	FKBP-type peptidyl-prolyl cis-trans isomerase involved in folding of proteins during protein synthesis. Contains calcium-binding EF-hand domain.

UPF0577 protein KIAA1324-like homolog (Q3UZV7)	Protein containing nine Cystein Domains of family 3 GPCR

Ovocalyxin-36 (Q53HW8)	Specific chicken eggshell matrix protein with antimicrobial activity Potentially involved in the mineralization of the eggshell. Contains LBP/BPI/CETP family, N-terminal domain

Glutamate [NMDA] receptor subunit zeta-1 (Q5R1P0)	Receptor family ligand binding region, Calmodulin-binding domain C0 of NMDA receptor NR1 subunit, Ligand-gated ion channel

Calnexin (Q5R440)	Molecular chaperone and calcium binding protein, which interact to newly synthesized glycoproteins to play a role in protein folding

Butyrophilin subfamily 1 member A1 (Q62556)	Specific membrane-associated receptor. Contains CD80-like C2-set immunoglobulin domain, B302 (SPRY) domain and Ig-like V-type (immunoglobulin-like) domain

UDP-glucuronosyltransferase 1-1 (Q63886)	UDP-glucoronosyl and UDP-glucosyl transferase family. Involved in detoxication and elimination of toxics

Mannose-binding protein C (Q66S61)	Binds mannose and N-acetylglucosamine in a calcium-dependent manner. Is capable of host defense. Contains Collagen triple helix repeat, Lectin C-type domain

Renin receptor (Q6AXS4)	Renin receptor-like protein

Avian Beta Defensin 9 (Q6QLR1)	Beta defensin family having bactericidal activties

Protein shisa-2 homolog (Q6UWI4)	Plays an essential role in the maturation of presomitic mesoderm cells by individual attenuation of both FGF and WNT signalling

Tissue factor pathway inhibitor 2 (Q7YRQ8)	Protease inhibitor (Kunitz/Bovine pancreatic trypsin inhibitor (BPTI) domain) involved in tissue modelling

Ganglioside GM2 activator (Q8HXX6)	Contains MD-2-related lipid-recognition domain involved in innate immunity and lipid metabolism

Dentin matrix protein 4 (Q8IXL6)	Calcium-binding protein, which may play a role in dentin mineralization

BMP-binding endothelial regulator protein (Q8N8U9)	Inhibitor of bone morphogenetic protein (BMP). Contains C8 domain, Trypsin Inhibitor like cysteine rich domain, von Willebrand (VWFC) factor type C domain and type D (VWFD) domains

78 kDa glucose-regulated protein (Q90593)	Heat shock protein (Hsp) 70 family. Probably plays a role in facilitating the assembly of multimeric protein complexes

Chordin (Q91713)	Involved in embryonic development via BMP2/4. Contains CHRD (chordin) and von Willebrand (VWFC) factor type C domains

EMI domain-containing protein 1 (Q91VF5)	Involved in tissue remodelling. Contains collagen triple helix repeat domain, EMI domain

Interleukin-17 receptor A (Q96F46)	Receptor for IL17A containing SEFIR domain

Calsyntenin-3 (Q99JH7)	Involved in cell adhesion, synaptic transmission, may modulate calcium-mediated postsynaptic signals. Contains cadherin domain

UPF0556 protein C19orf10 homolog (Q9CPT4)	Uncharacterised protein family UPF0556

Beta-amyloid protein 751 isoform (Q9DGJ7)	Protein containing Amyloid A4 extracellular, Beta-amyloid peptide, Kunitz/Bovine pancreatic trypsin inhibitor domains

Anthrax toxin receptor 1 (Q9H6X2)	Protein with Anthrax receptor C-terminus region, Anthrax receptor extracellular and von Willebrand factor type A (VWFA) domains

Thioredoxin domain-containing protein 16 (Q9P2K2)	Potentially involved in cell redox homeostasis

Ovocleidin-116 (Q9PUT1)	Chicken eggshell matrix protein constituting the major core of eggshell proteoglycan. May be involved in the mineralization process

Protein sel1 homolog 1 (Q9UBV2)	Sel1 family protein. May play a role in Notch signalling. Contains fibronectin type-II domain and Sel1-like repeats

Spondin-1 (Q9W770)	Cell adhesion protein containing Reeler, spondin-N and thrombospondin type 1 domains

Angiopoietin-related protein 3 (Q9Y5C1)	Fibrinogen beta and gamma chains, C-terminal globular domain protein

Beta-secretase 2 (Q9Y5Z0)	Aspartyl protease with broad endopeptidase specificity

**Table 3:** Biochemical properties of putative proteins secreted in the hen's uterus

Protein name	SwissProt Accession #	Isoelectric point	Aspartic acid (%)	Glutamic acid (%)	Arginine (%)	Lysine (%)
Neuroplastin	P97300	3.21	3.6	9.5	5.9	5.9

Calnexin	Q5R440	4.47	11.9	11.7	3.3	9.6

Osteopontin	P23498	4.53	12.9	9.3	6.5	4

Beta-amyloid protein	Q9DGJ7	4.65	7.5	11.7	4.5	5.3

Endoplasmin	P08110	4.81	7.8	13.6	4.4	10.3

Peptidyl-prolyl cis-trans isomerase	Q2KJC8	4.82	8.4	6.2	4.2	4.7

Beta-secretase 2	Q9Y5Z0	4.92	4	5.4	4.2	2.8

Nucleobindin-2	P80303	4.99	8.8	15.9	3.8	12.4

Interleukin-17 receptor A	Q96F46	5.06	5.7	7.5	5.7	2.4

Butyrophilin subfamily 1 member A1	Q62556	5.07	5.8	7.4	6	4.2

78 kDa glucose-regulated protein	Q90593	5.12	7.2	9.8	4.4	9.2

Thioredoxin domain-containing protein 16	Q9P2K2	5.13	5.3	8.4	3.6	6.5

Follistatin-related protein 1	Q12841	5.15	5.9	11.1	4.5	8.7

ICOS ligand	O75144	5.15	5	5.3	5.3	2

Protein sel-1 homolog 1	Q9UBV2	5.16	5.2	8	4.4	4.7

Calsyntenin-3	Q99JH7	5.17	5.9	7.8	4.8	3.9

Apolipoprotein A-I	P08250	5.26	6.2	13.3	7.5	9.6

Podocalyxin-like protein 1	O00592	5.28	4.7	5.7	2.4	5.1

Renin receptor	Q6AXS4	5.36	6	5.7	5.4	3.9

Ovocalyxin-36	Q53HW8	5.38	4.5	3	2.5	2.3

Ganglioside GM2 activator	Q8HXX6	5.38	4.8	7.2	3	6.6

Neuronal pentraxin-2	P47972	5.45	4.3	8.4	6	4.3

Beta-2-microglobulin	P21611	5.46	7.1	5.1	2	7.1

UPF0577 protein KIAA1324-like homolog	Q3UZV7	5.47	5.4	6.5	2.8	6.9

Glioma pathogenesis-related protein 1	P48060	8.7	5.7	2	4.1	6.1

UDP-glucuronosyltransferase 1-1	Q63886	8.87	3.8	5.1	4.3	6.3

Glutamate [NMDA] receptor subunit zeta-1	Q5R1P0	8.92	4.8	6.1	6.1	6.1

Avian Beta defensin-9	Q6QLR1	8.94	4.8	0	7.1	7.1

Glycine receptor subunit beta	P48167	9.03	5.4	4.2	4.4	7.8

Tissue factor pathway inhibitor 2	Q7YRQ8	9.06	4.2	6.6	6.6	10.8

EMI domain-containing protein 1	Q91VF5	9.17	2.4	5	6.4	3.1

Ovocalyxin-21	IPI00574331	9.3	3.23	5.16	7.1	3.87

Serum amyloid A protein	P02740	9.59	9.2	3.7	13.8	1.8

## Discussion

The eggshell is a sophisticated dynamic structure essential for successful reproduction of birds. Its architecture allows the diffusion of gases (O_2 _and CO_2_) between the developing embryo and the external environment. It also functions as a natural mechanical barrier to protect egg contents from the physical and microbial environment. Its integrity and strength is therefore critical for survival of the developing embryo and for consumers to ensure that table eggs are free of pathogens. The exceptional mechanical properties of the shell are the result of interactions between eggshell minerals (calcium carbonate) and organic macromolecules (proteins, glycoproteins and proteoglycans), which comprise the organic matrix, a key factor required for shell calcification [7,9,34]. Although the chicken eggshell is a very effective protective system, bacteria can penetrate the egg or enter the uterus *via *retrograde movement of fecal fluid from the cloaca prior to eggshell formation. Antimicrobial protection is a function that has been most commonly ascribed to numerous egg white proteins that possess antimicrobial properties [3,35], although this role was also described for the eggshell matrix. Partially purified eggshell matrix exhibits antimicrobial activity against *Pseudomonas aeruginosa*, *Staphylococcus aureus *and *Bacillus cereus *[36], which cannot be solely explained by the presence of lysozyme [11], ovotransferrin [12] and ovocalyxin-36 [22]--three principal antimicrobial proteins identified in the eggshell. In such a context, the identification and characterization of organic matrix components has stimulated numerous studies recently reviewed [7,34].

In the present study, we have used transcriptional profiling of the hen's oviduct to identify genes that are differentially expressed in the uterus during eggshell calcification. Egg proteins are sequentially deposited in the magnum, white isthmus and uterus as the forming egg passes through the hen's oviduct (Figures 1 and 2). The entire oviduct originates from the same population of cells [37], which specialize at sexual maturity into specific regions (magnum, isthmus and uterus) responsible for the deposition of egg white (magnum), eggshell membranes (white isthmus) and calcified shell (uterus) as the egg and its shell are formed. Consequently, the comparison of gene expression in the uterus where the eggshell is formed with two other segments of the oviduct (magnum or white isthmus) should reveal genes encoding proteins involved in supplying mineral and organic precursors that participate in eggshell formation. Using this unique approach, differential expression of genes should reveal specific functions of each specialized region that secrete egg components. Our study revealed a total of 605 highly expressed transcripts that correspond to 469 different genes (UniGene database) and 437 proteins. Forty-five transcripts have no match in nucleotide or protein databases and are considered as unknown genes present in the chicken genome.

Previous studies have shown that the organic matrix is made of unique proteins including ovocleidin-116 [18], ovocalyxin-36 [22], ovocalyxin-32 [21] and ovocalyxin-21 [8] (Figure 2). These four proteins are preferentially expressed in the uterus during eggshell calcification. A single cDNA insert corresponding to ovocalyxin-32 was present on our array but not expressed in the oviduct tissue. In contrast, the other three specific eggshell matrix genes were expressed only in the uterus as expected. Osteopontin (SPP1), a phosphorylated glycoprotein found in bone, kidney and various body secretions is over-expressed in epithelial cells of the uterus during eggshell calcification [13]. SPP1 was over expressed in the uterus in our microarray study as indicated by a 3.9- and 4.1-fold higher expression when compared to magnum and isthmus, respectively. Sixteen additional genes, over-expressed on microarrays were validated using qRT-PCR. Genes selected for qRT-PCR verification represent a wide range of fold differences (log2 ratios from 0.1 to 6.3) in uterine genes with low levels (10 to 41% higher), intermediate levels (52 to 100% higher), high levels (114% to 273% higher) and very abundant levels (up to 300% greater) in the uterus when compared to either the magnum or isthmus. From the 32 samples used in the microarray analysis, 31 laying hen oviduct samples were over-expressed in uterus. Only a single sample, corresponding to the lowest fold change (log2 ratio of 0.1), could not be validated by qRT-PCR.

There are few reports of global gene expression in chickens, while only one paper is related to the hen's reproductive tract [38], where oviduct gene expression was compared in mature *versus *juvenile birds using a custom 8 K cDNA microarray. Consequently, the over expressed genes were related to the dramatic changes due to the sexual maturity and the onset of egg production. In contrast, our samples were collected from mature hens during active calcification of the eggshell (i.e., 18 h post ovulation). Therefore, our transcriptional analysis was focused on the uterus (shell gland) during deposition of the eggshell. This approach allowed us to establish for the first time, the uterine transcriptome and 605 activated genes potentially related to eggshell deposition and associated cellular pathways. The functions of the 605 novel uterine transcripts were investigated using Gene Ontology (GO) annotation. The GO terms of the over-expressed genes in the uterus were compared to all GO terms represented on the 14 K array. The most over-represented proteins (GO terms) were related to ion transport which occurs during calcification [39,40]. Our transcriptional analysis has confirmed proteins previously identified as transporters and revealed new ionic transporters involved in supply of minerals needed for building the eggshell (Jonchère *et al*., in preparation). In addition, a GO term revealed a higher expression of proteins involved in synaptic transmission (Table 1). This observation could be related with the activation of muscle contraction and mobility of the uterus during rotation of the egg to facilitate calcification and/or final expulsion of the completed egg [41]. Our study has also demonstrated high abundance of genes involved in protein synthesis during the eggshell formation.

The uterus synthesizes both intracellular and extracellular proteins, which are secreted into the uterine fluid where the mineralization takes place. We paid particular attention to the extracellular proteins, which form the eggshell matrix and consequently are suspected to be involved in mineralization or chemical protection of the egg. Our first approach was to compare proteins encoded by uterine genes with those identified by proteomics. Indeed, proteomics is an important high-throughput methodology, which enabled the identification of 528 proteins in the calcified eggshell [25,26]. Our study confirmed uterine expression of 52 previously characterized eggshell proteins and transcripts for several new proteins not yet characterized in the eggshell. This limited number is partly due to the fact that some eggshell proteins are also expressed in other tissues along the oviduct. Consequently, these proteins are present in the eggshell, although but not revealed by our transcriptional analysis. The main advantage of the proteomics method is the ability to identify minute amounts of biologically active proteins in tissue or fluid. The eggshell proteome contains a complex mixture of uterine-derived proteins, additional proteins derived from degraded cells or basement membranes and those issued from the upper oviduct (i.e., egg white, egg yolk and vitelline membrane proteins) [25,42]. The number of eggshell proteins identified by mass spectrometry (528 proteins) is 4-5 times greater than those found in other egg compartments (i.e., 148 proteins in egg white, 137 in the vitelline membrane and 316 in egg yolk) [43-47]. Consequently, it is likely that the eggshell also passively incorporates proteins produced in the upper oviduct. To determine which proteins are potentially secreted by uterine cells and then deposited in the shell, we examined the presence of a signal peptide in 437 protein sequences obtained from the 605 highly-expressed uterine transcripts. A total of 54 proteins with signal peptide sequences were identified using several protein-centric databases (UniprotKB database, InterPro functional domain annotations, PubMed publications) (Table 2). These proteins were classified according to their biological function in the eggshell. The first group contains proteins involved in the biomineralization of the shell. For example, osteopontin [secreted phosphoprotein 1(SPP1)] is a protein found in both bone and eggshell [13]. The role of SPP1 in mineralization of the chicken eggshell has been described in detail [34]. Abnormal expression of *SPP1 *in the shell gland (uterus) is related to abnormalities and cracks in the eggshell [48]. Also included are ovocleidin-116 (OC-116), ovocalyxin-36 (OCX-36) and ovocalyxin-21 (OCX-21), which are three eggshell matrix proteins specific to uterine tissue [8,18,22]. Their presence is unique to the calcified shell and their expression limited to the uterus. OCX-21 contains a brichos domain and consequently, could play a role as molecular chaperone. A similar role is also proposed for endoplasmin (ENPL), a protein from the heat shock protein 90 family. Chaperone proteins in uterine fluid could play an important role in proper folding of the eggshell matrix, which is the crucial template for eggshell calcification. Several additional proteins involved in protein folding were identified in the 54 proteins possessing a signal peptide sequences. Among these, four proteins [ICOS ligand (ICOSLG), neuroplastin (NPTN), beta 2-microglobulin (B2M), butyrophilin subfamily 1 member A1(BTN1A1)] were previously identified in eggshell proteomic survey [25]. These four proteins contain immunoglobulin-like (Ig-like) domains involved in cell-cell recognition, cell-surface receptors and immune responses [49]. The Ig-like domain is one of the most common protein modules found in a variety of mammalian proteins including sandwich-like proteins, which are crucial for protein folding and conformation [50]. Lysosomal alpha manosidase (MAN2B1) plays also a role in protein folding and it is the most abundant uterine gene revealed by our microarray analysis. In the recent eggshell proteome survey [25], five proteins correspond to MAN2B1. MAN2B1 is a glycoside hydrolase, that participates in the metabolism of glycoproteins, maturation of N-glycans and in protein folding [51]. Its role is related to calnexin (CANX), an acidic protein (pI = 4.46) also identified as a putative uterine secretatory proteins, which have not been previously found among eggshell proteins. CANX is a molecular chaperone, which assists in protein folding. CANX binds only glycoproteins that have been folded by enzyme (i.e., MAN2B1). Consequently, these two proteins could be involved in metabolism of glycoprotein and proteoglycan, which are part of the eggshell matrix and thought to interact with calcite crystals and influence the texture of the mineralized shell and its mechanical properties [7,9,52].

SLIT, an axon guidance molecule involved in the embryonic development [53] was identified among our 54 secreted proteins (Table 2) and in the earlier eggshell proteomic analysis [25]. *SLIT2 *encodes a large extracellular matrix protein composed of leucine rich repeat motifs, which provide a structural framework for protein-protein interactions. In addition, SLIT protein contains a domain corresponding to epidermal growth factor (EGF) with a repeat pattern involving a number of cysteine residues thought to be important for the three-dimensional structure of proteins. Consequently, we believe that SLIT might be involved in folding of the eggshell matrix. It is also notable that SLIT has a calcium-binding site at the N-terminus of EGF-like domains. Calcium-binding properties often are a prerequisite for matrix proteins involved in calcium biomineralization. Consequently, SLIT could interact with calcium to favor crystal nucleation and morphology of crystals by interacting with some crystal faces of calcite. The ordered deposition of calcium carbonate (under the control of organic matrices) determines the texture of biominerals found in a large variety of calcified structures [39,54]. Amongst the 54 putative secretatory proteins, we have identified ten additional calcium-binding proteins; some of them were not previously characterized in the eggshell. These calcium binding proteins are endoplasmin (ENPL), SLIT2, SLIT3 (described above), nucleobindin-2 (NUCB2), follistatin-related protein-1 (FSTL1) and FK506-binding protein 9 (FKBP9); all contain calcium-binding EF-hand domains. Calcium is also a ligand of Calsyntherin-3(CLSTN3) and mannose-binding protein C (MBL2), which could also interact with calcium during eggshell fabrication. Another interesting secretatory protein is podocalyxin (PODXL), a sialoprotein, which was first identified in the renal glomerular podocytes [55] and more recently as a selectin ligand that facilitates metastasis [56]. Because of its high net negative charge, PODXL could interact with calcium carbonate during the calcification of the eggshell.

We also identified dentin matrix protein-4 (DMP4) as a secreted uterine protein. DMP4 is a calcium-binding protein that plays a role in dentin mineralization. This protein is a member of the FAM20 family corresponding to secreted proteins that regulate differentiation and function of hematopoiesis cells [57]. This protein was predicted as secreted and was found in the recent proteome survey [25]. We also paid a particular attention to BMP-binding endothelial regulator protein (BMPER) and chordin (CHRD). BMPER is a secreted protein known to interact with bone morphogenetic proteins (BMP-2, -4 and -6) and BMP2/4 antagonists in humans [58,59]. CHRD was first identified for its involvement in dorsalization of tissue in embryos. It is also a secreted protein, which binds BMP-2 -4 and -7 [60]. BMPs are members of the TGF-β superfamily of proteins and are known to induce the formation of new cartilage and bone following its ectopic implantation [61]. Studies in mollusks and coral suggest a role of BMPs in biomineralization [62-65]. Although BMP2 and BMP4 cDNAs were not present on our microarray, we used qRT- PCR to show higher level of expression (*P *< 0.02) of BMP2 in the uterus (0.686 ± 1.18) when compared to the magnum (0.034 ± 0.02). Therefore, it is likely that BMP2 is present in the uterine fluid and contributes to eggshell formation.

The second group of proteins, secreted in the uterus with a putative protective role, has antimicrobial properties. Antimicrobial proteins are found in the various compartments of the egg (yolk, egg white and shell), where they protect the egg against bacterial invasion, keeping the egg free of pathogens. Previous studies have shown that the eggshell matrix exhibits antimicrobial activity [36]. Three antimicrobial proteins (lysozyme, ovotransferrin and ovocalyxin-36) have been identified in the eggshell [11,12,22]. Our study has identified additional antimicrobial proteins secreted by the uterus, particularly proteins that contain Ig-like domains [ICOS ligand (ICOSL), neuroplastin (NPTN), beta-2-microglobulin (B2M), butyrophilin subfamily 1 member A1 (BTN1A1)], which are related to the immune responses [49]. Of particular interest are amyloid beta A4 protein (APP) and beta-amyloid protein 751 isoform (APP-751), which contain an amyloid extracellular domain and a heparin-binding domain. Heparin-binding proteins have basic domains, which might antimicrobial by binding to lipolysachharide (LPS) [66].

Our study has also revealed over-expression of avian β-defensin 9 (AvBD9) [previously called either gallinacin 9 or gallinacin 6] in the uterus. The avian β-defensins (AvBDs) are small cationic non-glycosylated peptides (1-10 kDa) with a three-stranded β-sheet structure connected with a β-hairpin loop that protect against gram-positive and gram-negative bacteria [5,67]. In mammals, β-defensins are involved in innate immunity and are capable of evading pathogen resistance mechanisms. In birds, AvBD9 is highly expressed in the trachea, esophagus and crop, while lower expression is found in skin, liver, testis and vas deferens [67]. Our transcriptional analysis indicates that *AvBD9 *is also expressed in the chicken uterus, where this antimicrobial peptide could contribute to the aseptic environment of the hen's oviduct. This idea is supported by the appearance of AvBD1-3 in cultured vaginal cells following *Salmonella *enteritidis or LPS exposure [68].

The third group of candidate proteins is proteases and antiproteases, which are involved in blood coagulation, cell migration and proliferation, innate defense and gamete maturation. We have identified three proteases: cathepsin A (CTSA, a serine carboxypeptidase), glioma pathogenesis-related protein 1 (GLIPR1, which contains a calcium chelating serine protease domain) and beta-secretase 2 (BACE1, an aspartyl protease). Previous work has shown that proteolytic activity present in uterine fluid varies according to the stage of the calcification [69]. Proteases could have a specific and controlled role during the calcification process, by either degrading proteins or regulating processing of proteins into mature forms. For example, CTSA has important roles in protein catabolism and in posttranslational processing of proteins and peptides, which ensures their stability and proper maturation [70].

Seven over-expressed genes encoding uterine antiproteases were identified in our study. Amyloid beta A4 protein (APP), follistatin-related protein 1(*FSTL1*), tissue factor pathway inhibitor 2 (*TFPI2*) and beta-amyloid protein 751 isoform (*APP-751*), all contain a Kunitz/Bovine pancreatic trypsin inhibitor domain. Alpha2-antiplasmin (*SERPINF2*) belongs to the serine protease inhibitor (or serpin) family. BMP-binding endothelial regulator protein (*BMPER*) contains a trypsin inhibitor like cysteine rich domain; and tissue metalloproteinase inhibitor 2 (*TIMP2*) belongs to the tissue inhibitor of metalloproteinase (TIMP) family. Proteases inhibitors could locally regulate the proteolytic activity of the uterine proteases or have an antimicrobial action by inhibiting bacterial proteases [71]. Besides their potential role in physical and chemical defense of the egg, the proteases and anti-proteases are likely to participate in embryonic development. The embryo gradually mobilizes calcium from the eggshell to ensure bone formation; therefore, active release of proteases or anti-proteases is needed for normal development. Interestingly, several proteases and anti-proteases identified in our work (i.e., APP, BACE1 and possibly APP-751) have been described in other species as major agents of neurite outgrowth and cell survival [72], whereas SERPINF2, TIMP2 and TFPI2 are implicated in angiogenesis and morphogenesis [73-75]. Additionally, FSTL1 is a regulator of early mesoderm patterning, somitogenesis, myogenesis and neural development in the chick embryo [76].

## Conclusions

Global gene expression profiling of the hen's oviduct during eggshell formation has revealed a large number of differentially expressed genes. Our study took advantage of tissue sampling from specialized segments of the oviduct that sequentially form different egg components and a bioinformatic analysis of the differentially expressed genes and their encoded proteins. This transcriptome approach enabled identification of more than 400 over-expressed genes in the uterus that are involved in providing precursors of the eggshell or proteins secreted into uterine fluid for fabrication of the eggshell and chemical protection of the egg. Our approach complements earlier focused proteomic analysis of the eggshell [25,26] that revealed more than 500 eggshell proteins, albeit less than 10% of the identified proteins were common to both strategies. The characterization of all proteins in the eggshell is a prerequisite for exploration of functional properties and regulation of uterine proteins involved in fabrication of the eggshell. Additional biochemical studies are needed to confirm the biological activity of these putative proteins and to understand their roles in providing nutrients and protection for the developing embryo. Our study could lead to improvements in the hygienic quality of this important human food and reveal novel proteins that might be useful for pharmacological applications. In addition, genes involved in the physical or chemical defense of the egg against pathological agents, are functional candidates for a marker assisted selection to improve egg and eggshell quality. Furthermore, identification of all protein components in the egg will allow optimization of the egg's defense system and, consequently, contribute to reduce risk of food-borne diseases.

## Methods

### Animals handling and housing

Brown egg-laying hens (ISA brown strain) were used in this study. The experiment was conducted at the Unité Expérimentale Pôle d'Expérimentation Avicole de Tours (UEPEAT - INRA, Tours, France) according to the legislation on research involving animal subjects set by the European Community Council Directive of November 24, 1986 (86/609/EEC) and under the supervision of an authorized scientist (Authorization # 7323). Forty-week old laying birds were caged individually and subjected to a light/dark cycle of 14 hr light and 10 hr darkness (14L:10D). The hens were fed a layer mash as recommended by the Institut National de la Recherche Agronomique (INRA). Each cage was equipped with a device for automatic recording of oviposition time.

### Collection of laying hens oviduct tissues

Tissues were collected from various regions of the oviduct (magnum, white isthmus and uterus) from mature laying hens. Tissue samples were harvested while the egg was in the uterus during the rapid phase of calcification (16-18 hr post-ovulation). Tissue samples were quickly frozen in liquid nitrogen and stored at -85 C until isolation of RNA.

### RNA isolation and microarray hybridization

The DEL-MAR 14 K Chicken Integrated Systems Microarray (NCBI GEO Accession # GPL1731) was constructed from 17,765 cDNA inserts, 387 long (70 mer) oligos and 72 quality control (QC) cDNAs [27]. The 14,053 unique cDNAs printed on our 14 K microarray represent 14,049 contigs and 3,716 singlets described in our original assembly of a chicken gene index [29]. This integrated systems microarray represents four major physiological systems with 9,833 unique cDNA clones from the metabolic and somatic systems and 7,937 unique cDNA clones from the neuroendocrine and reproductive systems [27]. Total RNA was extracted from frozen tissue samples using a commercial kit (RNeasy Mini kit, Qiagen; Courtabeouf, France) and simultaneously treated with DNase (RNase-free DNase set, Qiagen; Courtabeouf, France) according to the manufacturer's procedure. RNA concentrations were measured at 260 nm. The integrity of RNA was evaluated on a 1% agarose gel and with an Agilent 2100 Bioanalyzer (Agilent Technologies, Massy, France). Only RNA samples with a 28S/18S ratio > 1.3 were considered for labeling and hybridization. Twenty micrograms of total RNA were used for labeling the cDNA with the Superscript^® ^Plus Indirect cDNA Labelling System (Invitrogen, Cergy Pontoise, France). After synthesis and purification, the labeled cDNA sample was assessed with a Nanodrop ND 1000 (Nanodrop, Nyxor Biotech, Palaiseau, France).

A balanced block design was used for hybridization where half of the samples were labeled with Alexa^® ^555 fluorescent dye and the other half with Alexa^® ^647 (Fisher Scientific BioBLock, Illkirch, France). A total of 16 microarray slides were used for hybridization to 32 samples that correspond to two contrasts (uterus *versus *magnum; uterus *versus *white isthmus. The dye incorporation rate was estimated using a Nanodrop spectrophotometer (ND 1000, Palaiseau, France) and only cDNA probes with an incorporation efficiency of > 11.4 dye molecules/1000 bases were used for hybridization. All microarrays slides were prehybridized using 100 μL of DIG easy buffer (Roche Applied Science, Meylan, France) in humidified chambers for 1 hr at 42°C. Slides were then washed with distilled water for 10 min with mild agitation. An equal amount of Alexa^® ^555- and Alexa^® ^647-labelled cDNA probes from two samples was added to the hybridization solution (80 μl of DIG easy buffer, 2.5 μl of yeast tRNA (10 μg/μl, Ambion, Courtaboeuf, France), 2.5 μl DNA salmon sperm (10 μg/μl, Fisher Scientific BioBLock, Illkirch, France) and 2 μg PolyA RNA (1 μg/μl, Fisher Scientific BioBLock, Illkirch, France), then denatured at 100°C for 2 min. The mixture was loaded on slides, which were covered with Lifter^® ^cover slips (Erie Scientific, Portsmouth, NH) in hybridization chambers (Corning, Genas, France), then hybridized for 16 hr at 42°C. The slides were first washed in 0.2× saline sodium citrate (SSC) buffer and 0.1% sodium dodecyl sulfate (SDS) for 15 min at 42°C, then in 0.2× SSC for 15 min at room temperature. Finally, the slides were briefly rinsed with distilled water then centrifuged to dry. Microarray slides were scanned at 532 nm for Alexa^® ^555 and 635 nm for Alexa^® ^647 using a GenePix 4000 B microarray scanner (Axon Molecular Devices, Sunnyvale, CA, USA). GenePix Pro 6.0 software was used to acquire the fluorescent images, align the spots, quantify their intensity and finally export GenePix report (GPR) files containing spot intensity raw data. The GPR files were stored in the BioArray Software Environment (BASE) of SIGENAE (Système d'Information du projet d'Analyse des Genomes des Animaux d'Elevage) for further processing.

### Quantitative Reverse Transcriptase PCR (qRT-PCR)

Total RNA samples (5 μg) used for microarrays experiments were subjected to reverse-transcription using RNase H- MMLV reverse transcriptase (Superscript II, Invitrogen, Cergy Pontoise, France) and random hexamers (Amersham, Orsay, France). Classical PCR was performed using primers (Additional file 4) for 30 cycles at 60°C. Alternatively, cDNA sequences were amplified in real time using the qPCR Master mix plus for SYBR^® ^Green I assay (Eurogentec, Seraing, Belgium) with the ABI PRISM 7000 Sequence Detection System (Applied Biosystems, France). To account for variations in mRNA extraction and reverse transcription reaction between samples, mRNA levels were corrected relative to ribosomal 18S rRNA levels. The latter were measured using a TaqMan universal PCR master mix and developed TaqMan assay for human 18S rRNA (Applied Biosystems, Courtaboeuf, France) as previously validated [22]. The PCR conditions consisted of an uracil-*N*-glycosylase preincubation step at 50°C for 2 min, followed by a denaturation step at 95°C for 10 min, and 40 cycles of amplification (denaturation for 15 sec at 95°C, annealing and elongation for 1 min at 60°C). A melting curve was carried out from 60 to 95°C for each individual sample amplified with SYBR^® ^Green. Each run included triplicate of no template controls, control cDNA corresponding to a pool of uterine cDNA derived from laying hens sampled during eggshell formation and triplicate of samples. The threshold cycle (Ct), defined as the cycle at which fluorescence rises above a defined base line, was determined for each sample and control cDNA. A calibration curve was calculated using the Ct values of the control cDNA samples and relative amount of unknown samples were deduced from this curve. The ratio value was calculated for each sample as sample/18 S rRNA. The log of the ratio was used for statistical analysis using StatView version 5, software (SAS Institute Inc. Cary, NC). A one-way analysis of variance was performed to detect differences (*P *< 0.05) in gene expression in each region of the hen's oviduct.

### Statistical data analysis

Gene expression was compared between uterus and magnum (8 microarrays, 16 samples) and between uterus and isthmus (8 microarrays, 16 samples). For these two comparisons, differentially expressed genes were identified using the 'anapuce' package in R [77]. Spot intensities were calculated using the median value, which was transformed to log2 value. Normalization consisted of global locally-weighted regression (Lowess) applied on the overall intensity log2 ratio to remove dye bias due to efficiency of fluorescent dye incorporation. A block effect was corrected by subtracting the median value. Spot intensities were retained when present in at least 50% of samples. Assuming various sources of variance, we estimated the gene variance using a mixture model integrated into the VarMixt method [78]. Taking into account gene variance, we performed a unilateral statistical t-test to identify genes over-expressed in the uterus compared to either the magnum or white isthmus. P-values were adjusted by the Benjamini-Hochberg multiple testing procedures [79], to control false discovery rate (FDR<0.05). Statistical measurement of GO term enrichment were determined using an EASE score (*P *< 0.05), which is a conservative adjustment to Fisher exact probability [80].

The microarray data was deposited in the NCBI Gene Expression Omnibus (GEO) data repository under series accession number GSE17267 [81].

### Bioinformatics analysis of clones

The original annotation of cDNA clones used to produce our 14 K Del-Mar array was completed using CAP3 assemblies of 493 K chicken EST and cDNA sequences in GenBank [29]. A second annotation of differentially-expressed (DE) transcripts was performed using BioMart tool related to chicken contigs present in the SIGENAE database [30]. SIGENAE assemblies were carried out using chicken cDNA and EST sequences available in public databases. Resulting contigs were automatically annotated using BLASTX or BLASTN algorithms with different e-value cut-off, depending on used databases. These e-values were 10^-2 ^against UniGene and 10^-5 ^against UniProtKB. Expression Analysis Systematic Explorer (EASE) software [32] was used for automated functional annotation and classification of genes based on GO terms available in UniProtKB/Swiss-Prot databases. Functional annotation of the differentially expressed transcripts and putative proteins within the eggshell proteome [25] were performed using BlastX (E-Value cutoff 1 × e^-10 ^and minimum sequence identity of 65%).

The protein sequences depicted from the differentially expressed transcripts were analyzed for the presence of a peptide signal sequence using SignalP 3.0 [82]. Proteins were only accepted for secretion if both the neural network and hidden Markov model SignalP 3.0 algorithm identified presence of a signal peptide sequence, a cleavage site, and a Markov model probability higher than 95%. The biochemical properties of putative uterine proteins (pI and amino acid composition) were determined with Protparam software [83]

## Authors' contributions

VJ was involved in designing and planning of the study. He carried out the experiments and analyses, interpreted data, annotation and statistical analyses and wrote the first draft of the paper. SRG contributed to the interpretation of data, in defining biological roles of proteins and in the writing of the paper. CHA was involved in the experimental design, performed the statistical analysis, and contributed to the writing of the paper. CC developed bioinformatic tools used for annotation of genes and proteins and contributed to the writing of the paper. VS was involved in the experimental design, in preparation of data and contributed to the writing of the article. LAC developed the Del-Mar 14 K chicken microarray and was fully involved in design of the study and writing of the paper. YN conceived the research program focused on identification of egg proteins. He was involved in the experimental design, data interpretation and in the writing of the paper. JG is the supervisor of VJ (Ph.D. student). He conceived the strategy, designed and carried out experiments, interpreted data, annotation and statistical analyses and was fully involved in the writing of the paper. All authors have read and approved the final manuscript.

## Supplementary Material

Additional file 1 **Differentially expressed transcripts in uterus vs. magnum or white isthmus**. Excel file describing the differentially expressed transcripts in the comparison uterus vs. magnum **(**UT/Ma), uterus vs. white isthmus **(**Ut/WI) and 605 common genes between both contrast. Click here for file

Additional file 2 **Comparison of gene expression using microarray and qRT-PCR analyses**. Word file giving numerical and statistical data of the gene which were validated using qRT-PCR Click here for file

Additional file 3 **Correspondence between differentially expressed uterine genes and proteomic analysis of eggshell proteins**. The Excel file lists the uterine specific cDNA sequences and their correspondences with the eggshell proteins already reported, determined using BlastX. Exponentially modified protein abundance index (EmPAI) is an estimation of protein abundance based on the number of sequenced peptides in the proteomic study. Click here for file

Additional file 4 **List of primers used for RT-PCR**. Word file where are mentioned the primer sequences used in this study Click here for file
