# Supplementary material for: Gene expression profiling to identify eggshell proteins involved in physical defense of the chicken egg
Source: BMC Genomics. 2010 Jan 21;11:57. doi: 10.1186/1471-2164-11-57 (PMC2827412; doi:10.1186/1471-2164-11-57)
Supplement: Additional file 2 — Comparison of gene expression using microarray and qRT-PCR analyses. Word file giving numerical and statistical data of the gene which were validated using qRT-PCR [file 1471-2164-11-57-S2.DOC]

**Additional Table 1:** Comparison of uterine gene expression between microarray and

|  |  |  | **Uterus *vs.* Magnum** | | | **Uterus *vs.* Isthmus** | | |
| --- | --- | --- | --- | --- | --- | --- | --- | --- |
| **Clone name** | **Gene**  **symbol** | **SwissProt/ IPI protein IDs** | **Microarray** | **qRT-PCR** | | **Microarray** | **qRT-PCR** | |
|  |  |  | **log2 ratio** | **log2 ratio** | ***P*-value** | **log2 ratio** | **log2 ratio** | ***P*-value** |
| pgf1n.pk010.g12 | ***MAN1C1*** | Q9NR34 | 6.3 | 1.9 | 0.0265 | 2.1 | 0.4 | 0.4156 |
| pgr1n.pk002.d11 | *OCX-36* | Q53HW8 | 4.5 | 12.9 | <0.0001 | 4.4 | 7.8 | <0.0001 |
| pgl1n.pk011.j8 | *TXNDC16* | Q9P2K2 | 2.4 | 2.5 | 0.1184 | 1.5 | 1.7 | 0.8081 |
| pgf2n.pk005.f21 | *DMP4* | Q8IXL6 | 2.0 | 3.2 | 0.0001 | 0.9 | 0.5 | 0.2032 |
| pgp1n.pk006.d9 | *PODXL* | O00592 | 1.9 | 2.4 | 0.0428 | 1.1 | 0.4 | 0.3349 |
| pgp1n.pk001.h18 | *RCHY1* | Q96PM5 | 1.8 | 2.4 | 0.0265 | 1.0 | 0.7 | 0.4156 |
| pft1c.pk003.h9 | *FN1* | P07589 | 1.8 | 6.5 | 0.0060 | 1.0 | 4.4 | 0.2715 |
| pgf2n.pk006.i2 | *CALM1* | P62150 | 1.3 | 2.8 | 0.0240 | 0.8 | 1.0 | 0.0631 |
| pgm2n.pk010.i4 | *NPTN* | P97300 | 0.8 | 5.7 | 0.0007 | 0.4 | 2.3 | 0.1116 |
| pgp2n.pk003.k20 | *AAP* | P08697 | 0.7 | 4.9 | <0.0001 | 0.4 | 2.6 | 0.0013 |
| pgp1n.pk003.o11 | *CTSA* | P10619 | 0.7 | 2.1 | 0.0353 | 0.1 | -0.6 | 0.2986 |
| pgl1n.pk004.h14 | *BACE2* | Q9Y5Z0 | 0.6 | 3.7 | 0.0009 | 0.3 | 1.6 | 0.0911 |
| pgf1n.pk006.d19 | *CANX* | Q5R440 | 0.6 | 3.6 | 0.0430 | 0.4 | 1.5 | 0.1829 |
| pgp1n.pk008.o21 | *CLUSTN3* | Q99JH7 | 0.6 | 5.4 | <0.0001 | 0.3 | 3.2 | 0.0057 |
| pnl1s.pk003.c1 | *SAA* | P02740 | 0.5 | 1.9 | 0.0500 | 0.3 | 0.9 | 0.2651 |
| oligo40_603366341F1 | *OCX-21* | IPI00574331 | 4.0 | 9.0 | <0.0001 | 3.1 | 11.1 | <0.0001 |

qRT-PCR analyses
