# Supplementary material for: Gene expression profiling to identify eggshell proteins involved in physical defense of the chicken egg
Source: BMC Genomics. 2010 Jan 21;11:57. doi: 10.1186/1471-2164-11-57 (PMC2827412; doi:10.1186/1471-2164-11-57)
Supplement: Additional file 4 — List of primers used for RT-PCR. Word file where are mentioned the primer sequences used in this study [file 1471-2164-11-57-S4.DOC]

**Additional Table 2:** Primers used for qRT-PCR verification of uterine gene expression

| **Clone ID** | **Gene Symbol** | **RefSeq Accession #** | **Forward Primer** | **Reverse Primer** |
| --- | --- | --- | --- | --- |
| pft1c.pk003.h9 | ***FN1*** | XM_421868 | GCAAGCCAGTGATAGTGTAAGTCC | ACTCTTGAAAATGTCAGTCCCCC |
| pgf2n.pk005.f21 | ***DMP4*** | XM_414753 | CGAATGCTCTTACTACTGCTCAACC | TAGGAACGCCTCCAAGGGTTTCTC |
| pgf2n.pk006.i2 | ***CALM1*** | NM_001110364 | AAAGAGAATGTACTGGAGAGGCAGG | CCATGAGTGAACGACAGATAGATGG |
| pgp1n.pk001.h18 | ***RCHY1*** | AJ720511.1 | GCACGCAAATGGATGTATGCAG | TGAAGAGACACATGGAGTGGTGG |
| pgf1n.pk010.g12 | ***MAN1C1*** | XM_417735 | CCTTTATCCCAACTTTCTCAGCC | CCTTCTTGTCTGACATCAGCCAC |
| pgp1n.pk006.d9 | ***PODXL*** | XM_416475 | AAGCAGCATACACAAGCCAGGG | TCATGTGGTCTTTCAGGGTCGG |
| pgl1n.pk011.j8 | ***TXNDC16*** | XM_421472.2 | CTTTGCCACCATACCTTCGTTG | GGAGGAAATTGTAAGCAGGAAGAGG |
| pgp1n.pk008.o21 | ***CLSTN3*** | XM_416520.2 | TCGCACTGTTCCCAGCATTCAC | GCACAGGCAGCAAGTCAATCTC |
| pnl1s.pk003.c1 | ***SAA*** | AF289219 | CAATGTTGTCGAAGCCAACCAG | ACAAACTCACCCACCAAACACC |
| pgf1n.pk006.d19 | ***CANX*** | AJ719429 | TGAAATGGCTGCTGTATGTGACC | GGGACTGGTGCTCTGTAAGTAACC |
| pgm2n.pk010.i4 | ***NPTN*** | XM_413703.2 | TTAGCCTCTCCTGCAAGGATGACC | ATGCGTGATCCGACTTACTACTCC |
| pgl1n.pk004.h14 | ***BACE2*** | XM_416735 | AACTTGATGGTGAAACCTGGGAC | TTATGACGCAGAGTATTTGGAGGG |
| pgp1n.pk003.o11 | ***CTSA*** | AJ720789 | ACCGAGGTCGCACACAACAACTAC | CGATTCCCTTGAGGTTAAGGCTG |
| pgp2n.pk003.k20 | ***AAP*** | XM_41580 | AGAAATATGGTGTTGCTCTGGGG | CCACAGCTTTGTCTGAGGAGAGATG |
| pgr1n.pk002.d11 | ***OCX-36*** | NM_001030861 | TTGCAGTGCCATCCCTGTTC | CGGTCTGAATGATGGCATCG |
| oligo40_603366341F1 | ***OCX-21*** | XM_417666.2 | GCTGCAATTCTCATTTTGCTGGG | GAGTACAAGCCAGATCGGACAT |
| INRA cDNA clone | **BMP2** | NM_204358 | AAGGCATCCGTTGTATGTGG | AGCGGAAAAGGACATTCCC |
